# PET/CT in Multiple Myeloma: Beyond FDG

**DOI:** 10.3389/fonc.2020.622501

**Published:** 2021-01-25

**Authors:** Federica Matteucci, Giovanni Paganelli, Giovanni Martinelli, Claudio Cerchione

**Affiliations:** ^1^ Nuclear Medicine Unit, Istituto Scientifico Romagnolo per lo studio e la cura dei tumori (IRST) IRCCS, Meldola, Italy; ^2^ Hematology Unit, Istituto Scientifico Romagnolo per lo studio e la cura dei tumori (IRST) IRCCS, Meldola, Italy

**Keywords:** myeloma, choline positron emission tomography/computed tomography, 18F-fluorodeoxyglucose-positron emission tomography/computed tomography, new tracers, methionine positron emission tomography/computed tomography, multiple myeloma, FDG-PET/CT

## Abstract

Recent advances in the diagnosis and treatment of multiple myeloma (MM) have highlighted the importance of imaging methods, not only in the localization and extent of the disease but also in prognostic stratification and assessment of response to therapy. In this context, PET/CT, combining both morphological and functional information, is particularly useful in this pathology. The tracer mostly used is 18F-FDG, a glucose analog, which provides extremely accurate information with a sensitivity ranging from 80 to 100%. However, this tracer has some limitations, mostly related to the physiological uptake of FDG in the bone marrow and brain, which reduce its effectiveness. For this reason, some studies in the literature have evaluated the effectiveness of other PET tracers, which provide information on protein metabolism or the synthesis of metabolic plasma membranes, such as choline and methionine, as well as innovative radiopharmaceuticals, directed against receptors expressed by cells of myeloma, including tracers directed to the chemokine receptor. This review analyzes the characteristics and accuracy of non-FDG tracers in the management of patients with multiple myeloma.

## Introduction

Multiple myeloma (MM) is a neoplastic disease characterized by the uncontrolled clonal proliferation of plasma cells in the bone marrow. Bone involvement occurs in approximately two thirds of patients at diagnosis and in nearly all patients during their disease in the form of focal osteolytic lesions (1).

For this reason, imaging provides useful information in the detection of both intramedullary and extramedullary disease, both in the differentiation between solitary plasmacytoma (SP) and MM and finally in the predictive evaluation of the progression from smoldering myeloma (SMM) to active disease. The limitations of planar radiography, which has long been the examination of choice in these patients, have been largely due to the use in clinical practice of new imaging modalities, represented by computed tomography (CT), magnetic resonance imaging (MRI), and from positron emission tomography/computed tomography (PET/CT). At present, the role of PET/CT with 18F-fluorodeoxyglucose (18F-FDG PET/CT) in MM has reached an extremely significant level of evidence, so much so that it is considered a method of choice both in the diagnostic phase and for prognosis, as well as in the assessment of response to treatment. In particular, the role of 18F-FDG PET/CT has been extensively studied both in the diagnostic phase and in the prognostic evaluation of the disease and in the response to treatment, reaching very significant levels of evidence.

According to the update of the International Myeloma Working Group (IMWG), the presence of one or more osteolytic lesions evident on CT or PET/CT is indicative of bone disease, thus requiring specific treatment (2).

18F-FDG PET/CT represents a modality of choice in the various phases of MM: studies in the literature report a high sensitivity and specificity both in the evaluation of bone marrow and extramedullary disease, ranging from 80% to 100% (3–5).

In other studies, a comparison was made between the diagnostic performance of FDG-PET and MRI, highlighting how the sensitivity of FDG-PET is substantially equal to or slightly lower than that of pelvic-spinal MRI (PR-MRI) both in the evidence of diffuse infiltration of the spinal cord than in the visualization of focal lesions (6–10). In particular, Zamagni et al. (9) in a study on 46 patients showed how the FDG PET was not able to detect the infiltration of the bone marrow highlighted on MRI in 30% of patients, while the PET, performed in whole body, showed lesions located outside the MRI field of view in 35% of cases. The combination of the two methods allowed a correct diagnosis in 92% of the patients.

In patients with solitary plasmacytoma (SP), FDG-PET is instead able to detect additional lesions compared to MR, with greater sensitivity and specificity (8, 11).

In addition to its diagnostic value, FDG-PET has proved to be a fundamental tool for prognostic purposes, with undoubted usefulness in an era oriented towards precision medicine. In particular, in a study by Bartel et al. (12) found that the only imaging test significantly associated with an adverse prognosis for both overall survival (OS) and event-free survival (EFS) was FDG-PET when the number of focal lesions was greater than three at baseline. Furthermore, Fouquet et al. showed that the presence of at least two hypermetabolic lesions by FDG-PET was predictive of rapid progression to MM (13). Also in smoldering multiple myeloma (SMM), a positive FDG-PET in the absence of evident osteolytic lesions on transmission CT may be predictive of progression to symptomatic MM. In a series of 122 patients with SMM, Siontis et al. (14) showed that the probability of progression to MM within 2 years for patients with FDG-PET positive (uptake with or without lytic lesions on transmission CT) was 75%, *vs.* 30% for patients with negative PET.

Finally, PET/CT 18F-FDG is a reliable tool for evaluating therapy in MM. Studies published in the literature have shown that obtaining complete metabolic remission (CMR) on FDG PET/CT in an interim evaluation before or after autologous stem cell transplantation (ASCT) is associated with a better survival rate, especially in patients with a complete biological response (15, 16). For these reasons, the IMWG strongly advised to consider 18F-FDG PET/CT as the preferred imaging technique to assess response to therapy in MM (16, 17). Despite the promising results reported by several groups, however, there are currently no unambiguous standard interpretation criteria. In fact, in many studies the interpretation of the images is mainly based on semi-quantitative analysis and in others on visual evaluation or on both methods.

Recently Zamagni et al. (18) published a study that aimed to establish unique criteria to define the complete metabolic response (CMR) to PET after therapy in a subgroup of newly diagnosed MM patients eligible for transplantation. The results confirmed that Deauville score can also be used in this subgroup of patients and that liver background can be a useful reference to identify CMR on PET after therapy.

However, the use of 18F-FDG PET/CT is not exempt from certain limitations: in particular in relation to its metabolic characteristics, the 18F-FDG appears less sensitive in highlighting a diffuse infiltration of the bone marrow and in the visualization of the lesions of the cranial theca, given the physiological uptake of the tracer in the brain.

In addition, the uptake of 18F-FDG, as an analogue of glucose, can present both false positive lesions (due to inflammation, post-surgical areas, recent use of chemotherapy, fractures, etc.) that falsely negative lesions (in the presence of high levels of blood glucose, or following administration of high-dose steroids, etc.). To overcome the limitations of 18F-FDG, many other PET tracers have been proposed in patients with MM: the aim of our review is to provide an overview of the new non-FDG PET tracers currently used in the management of patients with MM.

## Old Tracers for New Indications: Choline and Methionine

Choline, a component of phosphatidylcholine, is an indicator of the synthesis of plasma membranes; its use in oncology is linked to the evidence that uptake is greater in proliferating cells in relation to the growth of plasma membranes. Choline PET imaging is used in clinical practice in prostate cancer diagnostics.

Methionine, labeled with 11C, is an amino acid PET tracer used mainly in oncological diseases of the central nervous system. The rationale for its use in MM is related to the evidence that radiolabeled amino acids show rapid metabolic absorption and incorporation into newly synthesized immunoglobulins.

Studies in the literature have highlighted a possible role of PET with both choline and methionine in the management of patients with MM.

In particular, the first experience, that have evaluated the use of choline in myeloma, is due to the Bologna group, which, following the occasional finding of a PET choline positive myelomatous lesion in a patient studied for prostate cancer, compared the diagnostic performance of PET with 11C-choline with those of FDG-PET (19).

The study, conducted in a small cohort of 10 patients at different times of the disease, showed a difference, although not statistically significant, in the average number of lesions detected in the two methods, with a consequent change in the management of these patients.

About 10 years later, Cassou-Mounat (20) studied 21 MM patients with both FCH-PET/CT and FDG PET/CT, showing a significant difference in the number of choline PET *versus* FDG positive lesions [8.1 *vs* 4.6 for FDG (p < 0.001)] with a higher target/background ratio.

The difference in uptake between 18FDG and choline does not yet find an exhaustive explanation even if several hypotheses have been formulated: in particular, the finding of high choline uptake and low FDG accumulation could be linked to a lower expression of hexokinase-2, enzyme that catalyzes the phosphorylation of FDG, preventing its back diffusion through the cell membrane (21).

In this study carried out on 221 patients, the authors showed a low expression of the gene coding for hexokinase-2, in PET false-negative cases (5.3-fold change, P < 0.001), so provides a possible explanation for this feature.

Furthermore, a heterogeneity of the accumulation of the different tracers in the same patient has been highlighted in several studies, suggesting the simultaneous presence of multiple spatially separated clones that coexist in the same patient and the need to use more than one tracer in some situations that are difficult to interpret.

At present, choline-PET is not used in clinical practice for the management of patients with MM and its possible inclusion in flow charts will only be possible after validation of diagnostic accuracy in larger prospective studies.

Similar considerations can also be made in relation to the use of PET with methionine in patients with MM: PET/CT with 11C-methionine seems to have better performance than 18F-FDG in detecting myeloma lesions even if the literature is very limited and therefore insufficient for the use of this tracer in clinical practice.

The first study comparing PET with methionine and 18F-FDG PET/CT was published in 2013 by Nakamoto et al. in 20 patients (15 with MM and 5 with plasmacytoma), reporting a greater sensitivity of PET with methionine than FDG (89 *vs* 78%) (22).

These results were amply confirmed by the work published by the Würzburg group in 2017 (23), which analyzed 78 patients (4 solitary plasmacytoma, 5 SMM, 69 MM symptomatic), reporting a significantly greater ability of MET-PET to highlight both medullary and extramedullary lesions than FDG PET/CT (respectively 75.6 *vs* 60.3%; p < 0.01).

The authors also highlighted that both MET-PET correlates with the number of intramedullary lesions highlighted in iliac crest biopsies to a greater extent than 18F-FDG PET/CT (Spearman’s r respectively equal to 0.832 and 0.635).

PET MET also appears to be superior to PET choline: the same authors recently published a head-to-head comparison study of 11C-methionine and 11C-choline for metabolic imaging of MM in 19 patients with a history of MM (n = 18) or solitary bone plasmacytoma (n = 1). The results obtained showed that MET-PET is more sensitive, detecting a greater number of intramedullary lesions in about 40% of patients (24).

## New Tracers

Molecular imaging has made significant progress in recent years with the development of innovative tracers that assess metabolic pathways other than those considered in the past or that evaluate the expression of specific plasmacellular receptors.

Currently, some specific biomarkers for plasma cell disorders have been studied for both diagnostic and therapeutic purposes: in particular the chemokine receptor 4 (CXCR4) and the differentiation cluster 38 (CD38).

In particular, it looks very interesting CXCR4, a G protein-coupled member of the chemokine receptor family (25), expressed on hematopoietic stem and progenitor cells in the bone marrow niche. CXCL12 (stromal cell-derived factor 1) binds to CXCR4 and forms various downstream signaling pathways, resulting in multiple responses necessary for tumor growth and development, including chemotaxis and gene transcription. The CXCL12/CXCR4 pathway is also involved in cell migration, the return of hematopoietic stem cells to the bone marrow, angiogenesis and cell proliferation. It has been shown that CXCR4 is overexpressed in MM (26), correlating both with progression and outcome of the disease (27).

Pentixafor is a peptide with high affinity for CXCR4 and represents an extremely promising ligand, in relation to its theragnostic characteristics: in fact it can be marked with both 68Ga, becoming an ideal PET tracer and with beta-emitting isotopes, such as 90Y or 177 Lu, becoming a therapeutic tracer.

**Table 1** summarizes the main studies available about the role of 68Ga-Pentixafor PET/CT in MM patients.

**Table 1:** 68Ga-Pentixafor PET/CT: Summary of analyzed papers.

Author	Year	N° pts	Pathology	Control	Principal results
Lapa et al. (28)	2017	35	MM	FDG-PET (in 19 pts)	Overall Pentixafor-PET positivity 23/35 (66%) Pentixafor-PET>FDG-PET in 4/19 pts (21%), FDG-PET>Pentixafor-PET in 7/19 (37%). In 8/19 (42%) patients, both tracers detected an equal number of lesions.
Pan et al. (29)	2020	30	MM	FDG PET	Pentixafor-PET positive in 28/30 (93.3%), FDG-PET positive 16/30 (53,3%) Diffuse bone marrow lesions (n = 17): Pentixafor-PET positive in 88.2%), FDG-PET positive in 29.4% Focal bone marrow lesions (n = 13): Pentixafor-PET positive in 92.3%, FDG-PET positive in 69.2%
Zhou et al. (30)	2020	10	SMM	FDG-PET MET-PET	MET-PET positive in 2/10 pts, Pentixafor-PET positive in 5/10 pts, FDG-PET negative in all pts. Correlation between BMPC infiltration rate and SUVmean in MET-PET and Pentixafor-PET; no correlation with FDG-PET.
Philippe-Abbrederis et al. (26)	2015	14	MM	FDG-PET	FDG-PET positive in 9/14 pts (64.3%), Pentixafor-PET positive in 10/14 pts (71.4%). Lesions comparable in 3 pts, Pentixafor-PET>FDG-PET in 7 pts, FDG-PET> Pentixafor-PET in 2 pts, FDG-PET and Pentixafor-PET complementary in 2 pts.

MM, multiple myeloma; SMM, smoldering multiple myeloma; BMPC, bone marrow plasma cells; FDG-PET, 18F-FDG PET/CT; MET-PET, 11C-Methyonine PET/CT; Pentixafor-PET, 68Ga-Pentixafor PET/CT.

In particular, Lapa et al. in 2017 (28) published a study on 35 patients with MM, who underwent 68Ga-Pentixafor-PET/TC for the evaluation of any radiometabolic therapy, comparing with [18F]FDG-PET/CT and laboratory data.

The results showed positivity to 68Ga-Pentixafor-PET/CT in 66% of the patients studied, in 8/23 (34.8%) with intramedullary disease, in 13/23 (56.5%) with both intra- and extramedullary lesions and in 2/23 (8.7%) with extramedullary lesions only.

The result of PET/CT was not correlated with the different myeloma subtypes or with other serological parameters. Positivity to 68Ga-Pentixafor PET/CT was instead a negative prognostic factor (OS 181 ± 41 d in PET positive patients; median OS in negative patients not reached).

In the 19 patients in whom a comparison with 18F-FDG PET/CT was possible, 68Ga-Pentixafor PET/CT was able to highlight a greater number of lesions in 21% of cases, while 18F-FDG PET/CT was superior in 7/19 (37%). In the remaining 8/19 patients (42%), both tracers detected an equal number of lesions (p = 0.018).

Based on the results obtained, albeit with the limitations linked to the retrospective nature of the study and the small size of the sample also subjected to the 18F-FDG PET/CT, the authors concluded that 68Ga-Pentixafor PET/CT could represent a useful tool for selection of patients to be referred to radiometabolic therapy and prognostic stratification, while no real benefit for diagnostic purposes is currently evident. Recently, some Authors have compared 68Ga-Pentixafor PET/CT and 18F-FDG PET/CT in patients with newly diagnosed multiple myeloma (29). In this retrospective study, conducted in 30 homogeneous patients with a recent diagnosis of multiple myeloma (7 pts in stage I, 4 in stage II, and 19 in stage III), a comparison was made between PET/CT with 68Ga-Pentixafor and 18F-FDG PET/CT, using both qualitative and semi-quantitative criteria.

The visual analysis of the images showed the positivity of 68Ga-Pentixafor PET/CT compared to 18F-FDG PET/CT in a greater number of patients (28/30 *vs* 16/30, respectively).

The semi quantitative parameters measured with 68Ga-Pentixafor PET/CT showed a significant correlation with the organ damage score (CRAB criteria), while the same correlation did not exist considering the semi-quantitative parameters of 18F-FDG PET/CT. Based on the results obtained, the authors concluded that the quantification of 68Ga-Pentixafor PET/CT could be a promising biomarker and superior to 18F-FDG PET/CT in the evaluation of the tumor burden of newly diagnosed MM.

However, the study certainly presented various limitations, mainly related to the lack of a correlation between the tumor burden highlighted on 68Ga- Pentixafor PET/CT and magnetic resonance imaging, which still represents the gold standard for the evaluation of widespread involvement of the bone marrow of the spine.

Recently, Zhou et al. (30) evaluated for the first time the role of 11C-Met PET/CT and 68Ga-Pentixafor PET/CT in 10 smoldering multiple myeloma patients, compared to 18F-FDG PET/CT.

The correlation between the percentage of plasma cell infiltration and the PET uptake, expressed by the mean SUV value, measured in the lumbar spine, was analyzed: the results showed a significant correlation of 11C-MET PET/CT and 68Ga-Pentixafor PET/CT, but not 18F-FDG PET/CT.

The authors therefore highlighted a greater sensitivity of 11C-Met PET/CT and 68Ga-Pentixafor PET/CT in the evaluation of bone marrow involvement in patients with SMM, suggesting studies in larger cohorts and prospectively the role of these methods in early identification of patients with high-risk SMM.

The theragnostic approach for individualized therapy today represents one of the main objectives in the oncology field: from this perspective, the development of a ligand of the CXCR4 peptide that can be labeled with α or ß- isotopes is extremely interesting.

The first studies have reported significant results, highlighting a good tolerance of therapy with high initial response rates (31, 32). Further future developments should include the study of therapy in patients with multiple myeloma in the early stages of the disease, alone or in combination with other conventional therapies.

A new frontier in the field of molecular imaging lies in the possibility of labeling antibodies with positron-emitting isotopes, in what is commonly defined as immuno-PET.

It is known that multiple myeloma cells express CD38, a transmembrane glycoprotein, which is the target of immunotherapy with Daratumumab.

The possibility of labeling daratumumab with positron-emitting radioisotopes such as Copper-64 (64Cu) and Zirconium-89 (89Zr) could therefore allow the creation of PET tracers ideal for MM imaging.

The studies currently present in the literature were carried out on animal models: in the only first-in-human phase I study in six patients, 89Zr-DFO-daratumumab PET/CT demonstrated an excellent ability to highlight known myeloma lesions as well as locations unknown to previous investigations carried out (33).

Obviously, prospective studies will be necessary to validate these first experiences, which however appear extremely promising for the use of this PET tracer, especially with the aim of identifying those patients with MM who could benefit from this immunotherapy.

## Conclusion

At present, PET/CT with 18F-FDG is recognized as a useful tool in the management of patients with MM both in the diagnostic phase and in the assessment of response to therapy and in the prognostic stratification of patients.

However, the method is not free from some limitations and for this reason several alternative PET tracers have been studied for the detection of MM. Some of these radiotracers have provided promising results, such as 18F-choline and 11C-choline, 11C-methionine, 68 Ga-pentixafor, and 89Zr-Daratumumab, but most studies are currently based on small patient cohorts and therefore the evidence will need to be validated in further prospective clinical trials.

## Author Contributions

FM and CC provided for bibliographic research and critical reading of the existing literature. FM and CC provided the draft. FM, CC, GP, and GM checked the paper. All authors contributed to the article and approved the submitted version.

## Conflict of Interest

The authors declare that the research was conducted in the absence of any commercial or financial relationships that could be construed as a potential conflict of interest.
